# Feather keratin hydrolysates obtained from microbial keratinases: effect on hair fiber

**DOI:** 10.1186/1472-6750-13-15

**Published:** 2013-02-18

**Authors:** Ana Lúcia Vazquez Villa, Márcia Regina Senrra Aragão, Elisabete Pereira dos Santos, Ana Maria Mazotto, Russolina B Zingali, Edilma Paraguai de Souza, Alane Beatriz Vermelho

**Affiliations:** 1 Department of General Microbiology, Institute of Microbiology Paulo de Góes, Federal University of Rio de Janeiro, Rio de Janeiro, Brazil; 2 Estácio de Sá University, Rio de Janeiro, Brazil; 3 College of Pharmacy, Federal University of Rio de Janeiro, Rio de Janeiro, Brazil; 4 Departament of Medical Biochemistry, Institute of Biomedical Sciences, Federal University of Rio de Janeiro, Rio de Janeiro, Brazil; 5 Biotechnology Center- Bioinovar, Federal University of Rio de Janeiro, Rio de Janeiro, Brazil

**Keywords:** Keratin, Hydrolysate enzymatic, Peptides

## Abstract

**Background:**

Hair is composed mainly of keratin protein and a small amount of lipid. Protein hydrolysates, in particular those with low molecular weight distribution have been known to protect hair against chemical and environmental damage. Many types of protein hydrolysates from plants and animals have been used in hair and personal care such as keratin hydrolysates obtained from nails, horns and wool. Most of these hydrolysates are obtained by chemical hydrolysis and hydrothermal methods, but recently hydrolyzed hair keratin, feather keratin peptides, and feather meal peptides have been obtained by enzymatic hydrolysis using *Bacillus* spp in submerged fermentation.

**Results:**

Keratin peptides were obtained by enzymatic hydrolysis of keratinases using *Bacillus subtilis* AMR. The microorganism was grown on a feather medium, pH 8.0 (1% feathers) and supplemented with 0.01% of yeast extract, for 5 days, at 28°C with agitation. The supernatant containing the hydrolysates was colleted by centrifugation and ultra filtered in an AMICON system using nano–membranes (Millipore – YC05). The Proteins and peptides were analyzed using HPTLC and MALDI-TOF-MS. Commercial preparations of keratin hydrolysates were used as a comparative standard. After five days the feather had been degraded (90-95%) by the peptidases and keratinases of the microorganism. MALDI-TOF mass spectrometry showed multiple peaks that correspond to peptides in the range of 800 to 1079 Daltons and the commercial hydrolysate was in the range of 900 to 1400 Da. HPTLC showed lower molecular mass peptides and amino acids in the enzymatic hydrolysate when compared with the commercial hydrolysate . A mild shampoo and a rinse off conditioner were formulated with the enzymatic hydrolysate and applied to hair fibers to evaluate the hydration, with and without heat, using a Corneometer® CM 825. The hydration was more efficient with heat, suggesting a more complete incorporation of hydrolysates into the fibers. Scanning Electron Microscopy showed deposits of organic matter in the junction of the cuticles that probably collaborates to the sealing of the cuticles, increasing the brightness and softness.

**Conclusions:**

These results show that the enzymatic method to produce keratin peptides for hair care products is an attractive and eco- friendly method with a great potential in the cosmetic industry.

## Background

**Figure 1:**
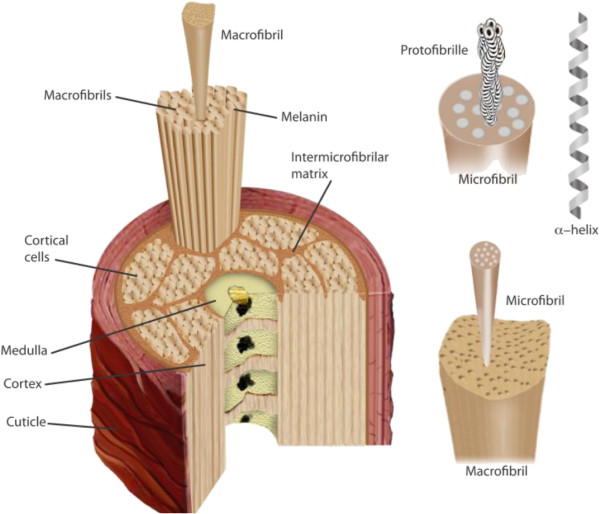
Hair structure.

Hair is composed mainly of keratin protein (90%) and a small amount of lipid (1–9%). The diameter of hair fibers varies between 40 and 150 μm and its major structure consists of a cuticle, cortex and medulla [1]. Most hair fiber mass is in the cortex which is responsible for the great tensile strength of hair fiber [2]. The cortex is made of long filaments packed together, named microfibrils which contain organized α-helical rods of keratin, embedded in an amorphous matrix [3,4]. The cortex is covered by an external cuticle, which accounts for 10% of the total weight of hair. The cuticle consists of overlapping layers of scales, each about 0.5 μm thick and is composed of β keratin. The cuticle protects the underlying cortex by acting as a barrier [5]. The medulla consists of specialized cells that contain air spaces. The medulla is frequently broken or missing from the hair shaft in fine hair [6]. The structure of hair is shown in Figure 1.

The major function of keratin cuticle is to protect the cortex of the hair from damage caused by several factors including heat, chemicals and daily maintenance. Keratin is a fibrous and insoluble protein with excellent mechanical properties. The chemistry of hair can also be modified by aging and by environmental factors such as pollution and sunlight [4]. Permanent waving, straightening or relaxing, bleaching during hair coloring processes and brushing can also cause damage to hair [6,7]. High concentrations of amino acid cysteine are responsible for its unique structure due to the strong chemical bond known as a disulfide bridge [8]. Keratin is found in other epidermic structures such as feathers, nails, scales and horns of mammals, reptiles and birds [9].

Protein hydrolysates are efficient restorers in hair care processes. These active peptides are reparative and conditioning agents and provide benefits for the hair such as strengthening hair fibers and reducing fiber breakages. Oligopeptides with a molecular mass <1,000 Daltons are able to penetrate the cortex. In permanent waving and bleaching, proteins have a substantial protecting effect on the hair structure. The addition of protein hydrolysates to hair coloring sprays and toners enables hair to absorb the dyes more uniformly. In the leave-on products, a natural conditioning effect of protein hydrolysates is reported [9,10]. Many types of proteic hydrolysates from plants and animals had been used in hair repair products and in also in skin cosmetics such as wheat protein [11] and keratin from nails, horns and wool [12]. Some commercial protein hydrolysates include Nutrilan H and Crotein A (hydrolyzed collagen), Elastin P (hydrolyzed elastin) and Crotein HKP S/F (keratin aminoacids) [13]. Most of them are obtained through chemical hydrolysis and hydrothermal methods. Recently hydrolyzed hair keratin [14], feather keratin peptides [15,16], and feather meal peptides [17] were obtained by our lab via enzymatic hydrolysis with *Bacillus* spp in submerged fermentation. The use of the enzymatic process to produce hydrolysates has grown immensely in recent years because it is more successful in preserving amino acids, and it is safer for the environment than the other methods and also it is a relatively gentle process [18].

The aim of this study was to produce keratin hydrolysates from feathers using microbial peptidases and then incorporate them into a cosmetic formulation for hair and investigate the effect of the treatment on hair fiber previously submitted to coloration, bleaching, relaxation and highlights.

## Methods

### Chemicals

Yeast extract was obtained from Oxoid Ltd. (Hampshire, England). Reagents used in electrophoresis were purchased from Amersham Life Science (Little Chalfont, England). All other reagents were of analytical grade. The molecular mass SDS–PAGE standards were obtained from Pharmacia Biotech. A commercial hydrolysate (KH1) obtained from chemically hydrolyzed animal keratin (hair and horns) was used as control.

### Protein content

This was determined in the culture supernatants according to Lowry et al. [19], using albumin bovine serum as the standard. Readings were carried out in a spectrophotometer at 660 nm.

### Feather keratin hydrolysates: enzymatic method

*Bacillus subtilis* AMR was inoculated in a medium containing yeast extract 0.5%, peptone 0.5%, KCl 2.0% and sucrose 2.0%. After 48 hours at 28°C the bacteria were washed three times with saline 0.85%. The inoculums (10^8^cell/ml) were added to 1 L of feather medium (yeast extract 0.01%, KCl 2.0% and supplemented with chicken feather 1%). The feathers were obtained from poultry waste and were previously washed with water and detergent, delipidated with chloroform: methanol (1:1, v/v) and dried at 60°C before use. The culture was grown for 5 days at 28°C/ rev min^−1^ in an orbital shaker. The crude feather keratin hydrolysate was obtained by centrifugation for 20 min at 2000 g. The supernatant containing the hydrolysates was concentrated further by ultrafiltration (Millipore) in an Amicon YC 05 system (1000 Daltons, NMWL, Nominal Molecular Weight Limit). The hydrolysates recovered corresponded to the enzymatic keratin hydrolysates.

### Keratinases and gelatinase assays

Keratinase assay was done as described by Mazotto et al. [14]. One unit of keratinolytic activity was defined as the amount of enzyme required to produce an increase of 0.01 absorbance unit, at 280 nm, under standard assay conditions (1 h at 37°C). Gelatinases were analyzed according the method described by Cedrola et al [16] One unit of gelatinase activity was defined as the amount of enzyme required to produce 1 lg of peptides under the described assay conditions.

### High-performance thin-layer chromatography (HPTLC)

The commercial and enzymatic keratin hydrolysates were placed on high-performance thin-layer chromatography plates (Merck silica gel 60 HPTLC). The development was carried out in butanol/acetic acid/distilled water (4:1:1 v/v/v) until the solvent front reached the top of the plate. The HPTLC plate was stained in ninhydrin reagent (7.5% in butanol/acetone 1:1 v/v). Commercial amino acids were used as the standard [15].

### Matrix-assisted laser desorption/ionization-time of flight (MALDI-TOF) mass spectrometry (MS)

The commercial and the enzymatic keratin hydrolysates obtained from feather fermentation by *B. subtilis* were identified using matrix assisted laser desorption/ionization time of flight mass spectrometry (MALDI-TOF MS). Immediately prior to mass spectrometry, acetonitrile/water (5:95 v:v) and trifluoroacetic acid were added to the samples. The sample was loaded using a hydrated Zip tip C18, after which it was washed with water. The sample was eluted three times with acetonitrile/water (60:40) containing 0.1% trifluoroacetic acid. An equal volume of α-cyano-4-hydroxycinnamic acid (CCA) matrix was added to the sample, and 1 μl of the sample mixture was spotted directly on a MALDI target for analysis. Peptide mass mapping was carried out with a Voyager DE PRO (Applied Biosystems) mass spectrometer [16].

### Cosmetic formulation with microbial keratin hydrolysates

A mild shampoo and a rinse-conditioner were prepared according to Tables 1 and 2. The hydrolysates were added at a concentration of 10% corresponding to 15,34 mg according to the protein measurement by the Lowry method (1534.86 μg/ml).

**Table 1:** Mild Shampoo composition - enzymatic hydrolysates from chicken feathers

**Components**	**% (P/P)**
Sodium lauryl sulfate	30
Decyl polyglucose	5
Laureth Decyl polyglucose	5
Surfax acid	3
Coconut fatty acid diethanolamide	4
Phenochem or Phenova	0.5
Germall 115	0.2
Keratin hydrolysate	10
Unistab 569	0.2
Essence of anise	0.5
Distilled water qs	100 ml

The Germall requires previous heating (80°C) until complete dissolution in distilled water. After this procedure all the other components were added to the solution. However the polyglucose must also be heated before incorporation into the solution. The mixtures should be homogenized slowly to avoid foaming. The last components to be added were the enzymatic hydrolysate from feathers, the Unistab 569 and the essence.

**Table 2:** Rinse-conditioner composition - enzymatic hydrolysates from chicken feathers

** Components**	**% (P/P)**
**Oily Phase**	
Cetearyl Alcohol	5
Phenova	0.5
Cetrimonium chloride	0.5
**Aqueous Phase**	
Germall 115	0.2
Essence of anis	0.5
Color	0.2
Keratin Hydrolyzed	10
Distilled water qs	100 ml

All components of the oily phase were heated (75°C) together and homogenized until complete dissolution. In another container, the Germall was dissolved by heating to 80°C. After reaching the required temperatures, the oily phase was added to the water phase, under agitation, until fully emulsified. The solution obtained was placed in a cold bath, homogenized and the other substances of the aqueous phase were then added.

### Cosmetic evaluation scheme

The treatment (mild shampoo and a rinse-conditioner containing the enzymatic keratin hydrolysate) was applied over a 5 week period to locks of virgin and chemically treated hair. The hair had been washed and defatted previous to the treatment with the shampoo and rinse-conditioner prepared with the microbial keratin hydrolysate. Then the samples underwent hair-drying and hair straightening at 180°C, in order to assess the degree of capillary fiber hydration.

**Figure 2:**
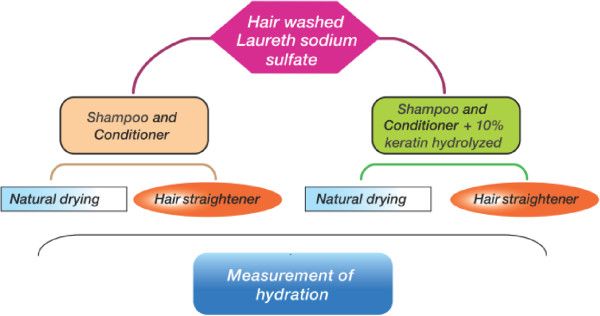
Flowchart to apply enzymatic hydrolysates on hair.

The hair locks were divided into 5 different groups: untreated hair, colored hair, colored and relaxed hair with thioglycolic acid, brown colored hair with blonde highlights and hair after bleaching. Each group was composed of four hair locks: two with 10% keratin hydrolysate (corresponding to 15,34 mg of protein) and two hair locks for control. Before the tests all the hair locks were washed well with sodium laureth sulfate 2% shampoo and rinsed with distilled water. This procedure was intended to remove any adsorbed material to avoid interference in the trial. Figure 2 is the flowchart of the treatment process.

The hydration measurement assays were carried out every 7 days using a Corneometer® CM 825 (Courage and Khazaka, Germany), which was mounted on a Multi Probe Adapter® MPA 5 (Courage and Khazaka, Germany). The measurement was made using the capacitance method. This method makes use of the relatively high dielectric constant of water (ε_r_ =81C^2^/Nm^2^) compared to other substances in the skin (ε_r_ <7C^2^/Nm^2^). The front surface of the sensor contains a measuring condenser. The capacitances change depending on the water content and these differences can be measured and converted into a digital value that is proportional to the skin or hair humidity. Because of the short measurement time, errors due to skin deformation or hair malleability or evaporative build-up were excluded. Ten measurements were made at different points of the hair treated with or without the peptides. The measurements were performed at a room temperature of 18-20°C and at a relative humidity ranging from 25% to 40%. The results are given in “arbitrary units” (A.U.). The Analysis of Variance ANOVA was used to statistically evaluate the final hydration of the hair structures.

### Scanning electron microscopy (SEM)

To characterize the effect of the keratin hydrolysate applied onto normal and chemically treated hair the samples were observed in a scanning electron microscope before and after the treatment. Samples were put on stubs and gold sputtered and then observed in a JEOL JSM 5310 scanning electron microscope operating at 15 kV [14].

## Results and discussion

**Figure 3:**
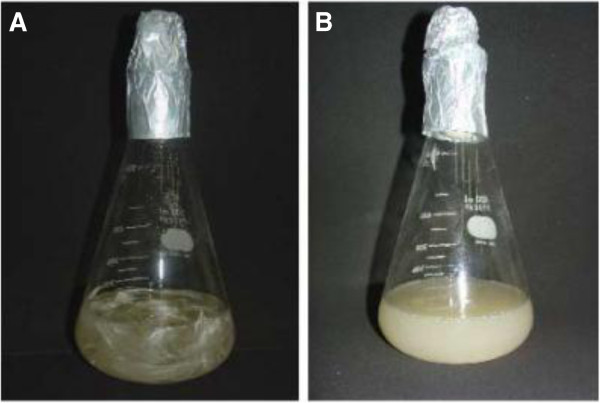
**A Control: *Bacillus subtilis *in feather containing medium (time 0) and B After 5 days of growth in feather medium.**

The biocatalytic uses for enzymes have grown immensely in recent years since they are ecologically correct, have a high specificity, present chemo-regio-enantio-selectivity and present a wide diversity of reactions. Moreover, the conditions to obtain and optimize the production of enzymes in terms of nutrients, pH, temperature and aeration are easily controlled in bioreactors. Microorganisms can also be manipulated genetically to improve the desirable characteristics of a biocatalyzer. These characteristics have encouraged the ever growing search for biocatalytic processes [20]. The goal of our study was to use an enzymatic process to obtain keratin hydrolysates for hair care products. Currently, the commercial keratin hydrolysates are obtained by chemical hydrolysis. The proposed method is environmental friendly and produces a clear hydrolysate. In contrast, the commercial hydrolysates have a dark color due to the presence of acid. The clear color is an advantage when incorporating keratin hydrolysate into products for hair or skin cosmetics. Whey proteins with a molecular mass lower than 10 kDa are characterized by reduced allergenicity. Therefore it is desirable to obtain fractions with molecular masses below 5 kDa in the hydrolysis process [21]. In addition Eremeev et al [22] demonstrated the antioxidant activity of keratin hydrolysates. The first step in this work involved transforming feathers into keratin peptides and amino acids by peptidases and keratinases produced by *Bacillus subtilis.* Figure 3 shows that the feathers were degraded (90-95%) by the microorganism after five days of growth in the medium. The keratinases and peptidases can act on other keratin residues including wool and horn powder. Keratinases are being applied in the feed, fertilizer, detergent, leather and pharmaceutical industries [15,16,18,23].

Some reports have described the production of keratinases by *Bacillus* species such as *B. subtilis* KD-N2 [24]; *B. pumilus* KS12 [25], *B. megaterium* SN1 [26]. However these works describe the isolation of new strains, mutant production and the characterization of keratinases suggesting its potential applications. In our study the focus was on the feather keratin hydrolysate produced by *B. subtilis,* specifically the peptides, and the aim of our work was to analyze the effect of the hydrolysate on hair fiber. Different methodologies for keratinases analysis have been used by other authors and this great variability makes it difficult to compare results. However the native strain of *Bacillus subtilis* used in the present manuscript showed an excellent proteolytic (gelatinase) activity with a production of 350 U/ml and 400 U/ml of keratinases and proteases respectively.

**Figure 4:**
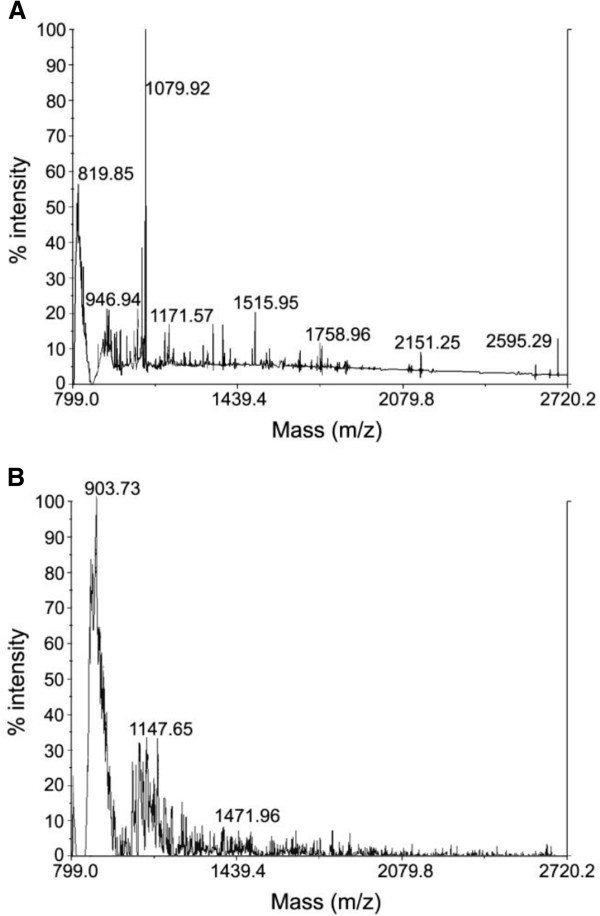
**MALDI-TOF MS analysis of the enzymatic keratin hydrolysates from feather keratin by *Bacillus subitilis *(A) and a commercial hydrolysate (KH1) (B).** For details see Materials and Methods.

The keratin peptides formed by enzymatic degradation were analyzed by Matrix-assisted laser desorption /ionization –time of flight (MALDI-TOF) mass spectrometry. First of all by comparing the two spectra we can observe the distinct profiles of the two hydrolysates. The multiple peaks corresponding to the peptides with a low molecular weight predominantly in the range of 800 to 1079 m/z were produced by *Bacillus subtilis* (Figure 4A). We also can observe some ions with m/z in the range of 1171.57 to 1758.96. While for the commercial keratin hydrolysate preparation KH1, the peaks were concentrated in the range of 900 to 1400 m/z as shown in Figure 4(B). Multiple peaks between 1400 and 2100 can also be seen in the same Figure. Thus the hydrolysate produced by *B. subtilis* contains peptides with lower molecular masses. These low molecular peptides can penetrate into the hair fiber more efficiently and this characteristic is a positive difference compared with the commercial hydrolysates. A previous work by our group using human hair as substrate demonstrated multiple peaks from 816 to 2080 m⁄z after 4 days of culture [14]. The molecular mass analysis of the culture supernatant produced by the *B.subtilis* strain SLC using feathers as substrate revealed that most peptides, derived from chicken feathers, presented a molecular mass in the range of 500-3000 Daltons [16]. In a thermophilic *Meiothermus ruber* H328, the MALDI TOF analysis of solubilized products after growth on feather medium detected only oligopeptides with less than 1,000 Daltons [27]. These results confirm that microbial enzymes produce peptides with a lower molecular mass.

**Figure 5:**
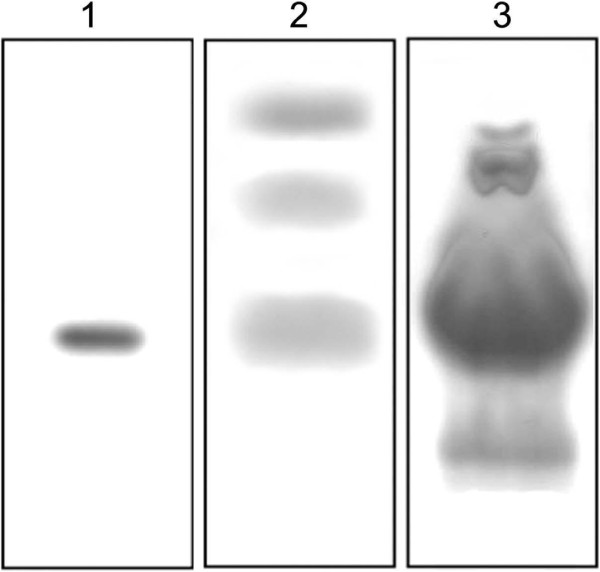
**HPTLC analysis of keratin peptides after filtration by ultrafiltration in the Amicon system (Millipore, 1000 Daltons). 1** - Amino acid glycine. **2** - Feather keratin peptides obtained by enzymatic hydrolysis. **3** - Commercial hydrolysate (KH1).

A preliminary analysis of the enzymatic keratin hydrolysates was done using thin-layer chromatography (HPTLC) and peptides and amino acids with a lower molecular mass were observed as shown in Figure 5, lane 2, when compared with the commercial hydrolysate (KH1) in lane 3. The amino acid glycine in lane 1 was used as a standard.

After fermentation, the enzymatic hydrolysate had a protein concentration of 3,5 mg/ml. After filtration the protein content went to1,5 mg/ml, corresponding to a percentage of 42, 8% relative to the total protein. The enzymatic hydrolysate was applied to the hair locks at a concentration of 10% as described in Materials and Methods and in Figure 2.

The hydrolysate was applied using a mild shampoo and a rinse-conditioner prepared according to Tables 1 and 2. Table 3 demonstrates that there is an increase in hydration for all hair treated with the enzymatic hydrolysates and with straightener at 180°C. Without the application of heat the process was not efficient, suggesting that heating is important to incorporate the hydrolysates in the hair.

**Table 3:** Hydration effect of the enzymatic hydrolysates on hair fiber

**Hair types**	**Types of Treatment (average – 5 weeks in AU)**				
	**A**	**B**	**C**	**D**	**E**
1. Untreated hair	7.363	8.236	8.108	7.454	7.836
2. Colored hair	7.181	8.145	8.126	7.017	7.654
3 Colored hair with highlights	7.09	7.781	7.526	7.017	7.29
4 Colored hair with relaxer	6.454	7.761	6.563	7.09	6.399
5.Bleached hair	7.181	7.672	7.563	7.036	7.326

A- start up hair (washed with sodium laureth sulfate) and dried naturally, B- hair washed with shampoo and rinse containing the enzymatic hydrolysates and dried with a straightener at 180ºC, C - hair washed with shampoo and rinse containing hydrolysates and dried naturally, D – control hair (washed with shampoo and rinse without hydrolysates) and dried with a straightener at 180ºC; E - control hair (washed with shampoo and rinse without hydrolysates) and dried naturally.

Protein hydrolysates, in particular those with a low molecular weight distribution-i.e., < 1,000 Daltons are known to provide efficient protection and care to hair. Various sources of proteins have been used to produce hydrolysates. Wheat protein [28], wool keratin [29] and collagenous hydrolysates [30] are examples that have been used in skin and personal hair care products and are known to confer improved compatibility, feel, moisturization and help maintain the natural structure [12,29]. In hair care products, the lower molecular weight peptides have two effects: 1) They are capable of penetrating the cortex of the hair fiber and 2) They can promote a surface coating. The penetration appears to be deeper with longer treatments. Besides this, bleached hair shows a higher level of penetration of hydrolysates when compared with non-damaged control hair [10]. These properties have beneficial effects on the hair structure replacing lost keratin and also have an antiaging effect [2]. The effect of wool keratin peptide on the skin in an aqueous or in liposome formulation was investigated by [12] and an increase in hydration and elasticity as a result of the keratin peptide application was observed.

**Figure 6:**
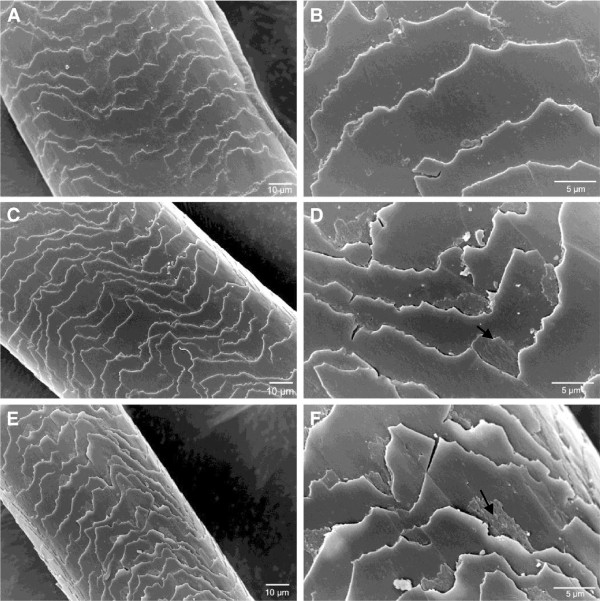
**Scanning electron microscopy (SEM) analysis of colored hair A, B –Control; C, D - After the treatment with the enzymatic hydrolysates and straightener at 180°; E, F- After the treatment with the enzymatic hydrolysates without heat.** Arrows indicate feather enzymatic hydrolysate deposits.

**Figure 7:**
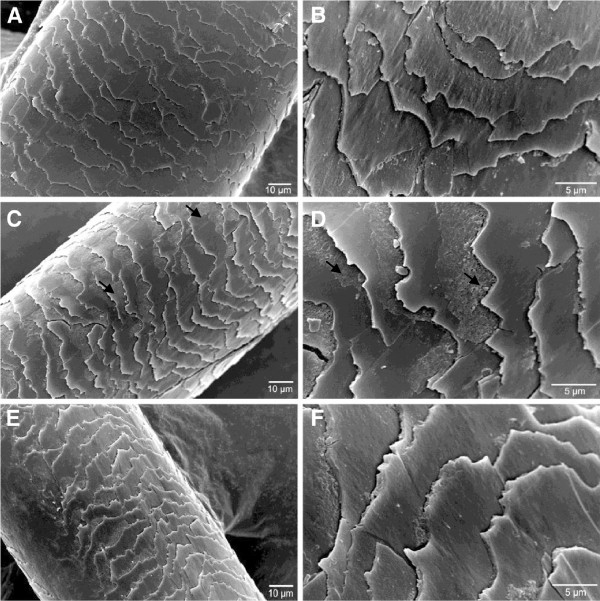
**SEM Micrography analysis of colored and straightened hair after enzymatic hydrolysate treatment.** Treated hair **A, B** - Control; **C, D**- After treatment with the enzymatic hydrolysates and straightener at 180°; **E, F**- After treatment with the enzymatic hydrolysates without heat, Note the deposit of the enzymatic hydrolysates on the scales (arrow).

**Figure 8:**
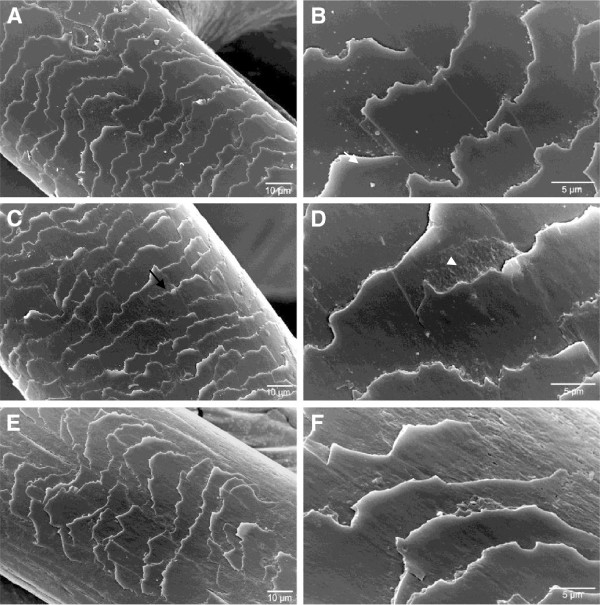
**Electronic scanning microscope images obtained from an untreated bleached hair (A, B) and treated with the enzymatic hydrolysates with heat (C,D) or without heat (E,F).** Black arrow indicates deposit of peptides from the enzymatic hydrolysates and white arrow shows the broken edges of the cuticle.

The effect of applying the enzymatic hydrolysate on hair fiber was evaluated by SEM (Figures 6, 7 and 8). All Figures show micrographs of hair fibers treated and untreated with the enzymatic hydrolysate. An increase in the brightness and softness was observed by sensory analysis (data not shown). However in the micrographs, deposits of feather keratin hydrolysates were observed in the junction of the cuticles of all hair types. This deposit probably collaborates to the sealing of the cuticles. Also heat is essential for the complete sealing of the cuticles. Colored fiber was benefited by the hydrolysates when applied with heat (Figure 6C, D). Hydrolysates adhered more to the previously colored and straightened hair fibers (Figure 7C, D) indicating that the combination of coloration and straightening favors the action of the keratin peptides. Figure 8(A, B) shows that the bleaching treatment promoted accentuated damage to the hair fiber. The appearance of the leading edge of the cuticle scales indicates a breaking up of the scales (B). The application of the enzymatic hydrolysates with heat collaborated for the sealing of the cuticles but their edges remained broken (C, D).

When hair chemistry is modified, some of the natural properties of hair are compromised. Several mechanisms can cause damage to hair fiber. For example, environmental stresses and UV radiation photo-oxidizes proteins. Protein photo-oxidation leads to the cleavage of disulfide bonds which cross-link the proteins, and breaking of thioester bonds, which results in the release of bound surface lipids and loss of hair structure. These reactions lead to a deterioration of hair properties, noticeable to consumers in the form of poor manageability, dryness and brittleness, loss of shine and, in extreme cases, decreased strength [2]. Some cosmetic treatments like permanent curling, permanent coloring, bleaching and relaxing/straightening are known to alter hair properties [9,31]. Even cosmetic handling such as daily combing and brushing can damage hair [32]. Recently, Cao et al [33] used different concentrations of the fermentation broth (chicken feathers) obtained from *Stenotrophomonas maltophilia,* in the hair. The supernatant was incubated for 30 min. The broth was found to be protective to hair, as evidenced by the improved flexibility and strength for both normal and damaged hair.

Sionkowska, et al [9] using UV–Vis spectroscopy, Fourier transform, infrared spectroscopy (FTIR) and fluorescence spectroscopy, evaluated the influence of UV irradiation on keratin hydrolysates. New photoproducts were formed during UV irradiation of keratin hydrolysates and a slight increase in oxidized sulfur species was also observed. The authors proposed that photodegradation of keratin hydrolysates could be a useful method for the preparation of hydrolysates with lower molecular weight. In the present work an increase in the hydration, brightness and softness was observed in the different types of hair after the treatment with 10% of keratin peptides obtained by the enzymatic hydrolysis process. The use of feathers, an industrial waste generated by poultry as a biomass source for the process is very interesting because this raw material is cheap and it is bio transformed into a new product with an aggregate value. Currently, keratin hydrolysates are usually prepared from keratin-containing animal parts, such as feathers, horns, hoofs, hair and wool. Aromatic amino acids (tryptophan, tyrosine, and phenylalanine) and cystine (amino acid containing sulfur) play a pivotal role in the photochemistry of keratin [8]. Some industries have developed products which use a complex of 18 free amino acids derived from wheat, corn and soy proteins to mimic the natural composition of keratin. The high sulfur amino acid content of the soy is similar to that of human hair and wool [7]. However, keratin is an irreplaceable protein in respect to its mechanical and protective properties.

The enzymatic method described in the present work can be used for industrial wastes/residues in general to produce value added products**.** Previous studies in the literature have described the use of keratinases /peptidases for recycling feather keratin discarded by the poultry industry [34-36]. The present work reports for the first time on the use of keratin peptides in the cosmetic industry, specially focused on the hair care segment. Taking into consideration all these factors, the enzymatic method for keratin peptide production for hair care products is an attractive and eco- friendly method with great potential within the cosmetic industry.

## Conclusion

Our results demonstrate that the keratin hydrolysates obtained enzimatically are peptides with a molecular mass of 800 to 1079 Daltons. The keratin peptides increased the hydration of hair fiber and scanning electron microscopy analysis showed sealed cuticles in the fibers treated with the hydrolysates, which also presented a significative increase of the brightness and softness.

The enzymatic hydrolysis is an attractive method for biotechnological applications in the cosmetic industry, taking into consideration that it is an eco-friendly method, based on the sustainability and in the biotransformatiom of a protein rich biomass.

## Competing interests

The authors declare that they have no competing interests.

## Authors’ contributions

ALV and ABV designed the experiments and wrote the manuscript. ALV, MRA, AM and EPS carried out the experiments, EPdS, developed the cosmetic formulations, and RLZ did the MALDI TOF analysis. All authors have read and approved the final manuscript.
